# The role of extracellular histone in organ injury

**DOI:** 10.1038/cddis.2017.52

**Published:** 2017-05-25

**Authors:** Eleanor Silk, Hailin Zhao, Hao Weng, Daqing Ma

**Affiliations:** 1 Anaesthetics, Pain Medicine and Intensive Care, Department of Surgery and Cancer, Faculty of Medicine, Imperial College London, Chelsea and Westminster Hospital, London, UK; 2 Department of Anesthesiology, Shanghai Fengxian District Central Hospital, Shanghai Jiao Tong University Affiliated Sixth People’s Hospital South Campus, Fengxian District, Shanghai, China

## Abstract

Histones are intra-nuclear cationic proteins that are present in all eukaryotic cells and are highly conserved across species. Within the nucleus, they provide structural stability to chromatin and regulate gene expression. Histone may be released into the extracellular space in three forms: freely, as a DNA-bound nucleosome or as part of neutrophil extracellular traps, and all three can be detected in serum after significant cellular death such as sepsis, trauma, ischaemia/reperfusion injury and autoimmune disease. Once in the extracellular space, histones act as damage-associated molecular pattern proteins, activating the immune system and causing further cytotoxicity. They interact with Toll-like receptors (TLRs), complement and the phospholipids of cell membranes inducing endothelial and epithelial cytotoxicity, TLR2/TLR4/TLR9 activation and pro-inflammatory cytokine/chemokine release via MyD88, NFκB and NLRP3 inflammasome-dependent pathways. Drugs that block the release of histone, neutralise circulating histone or block histone signal transduction provide significant protection from mortality in animal models of acute organ injury but warrant further research to inform future clinical applications.

## Facts

- Histone is a highly cationic intra-nuclear protein that supports the normal structural development of chromatin and regulation of gene expression.
- Histone and DNA-bound histone may be released into the extracellular space during cell death processes including necrosis, apoptosis and NETosis.
- In acute sterile organ injury, cell death occurs due to various toxic stimuli including ischaemic, traumatic and autoimmune pathology.
- In the extracellular space, histones act as cytotoxic damage-associated molecular pattern (DAMP) proteins by activating Toll-like receptors (TLRs), promoting pro-inflammatory cytokine pathways and altering phospholipid membrane permeability.
- In animal models of acute organ injury (AOI), anti-histone monoclonal antibodies and endogenous molecules (C-reactive protein and activated protein C) provide significant protection from mortality.

## Open Questions

- How can we accurately delineate the effects of free histone versus DNA-bound histone?
- How do different histone subtypes affect different tissue types? Does cytotoxicity vary by histone subtype in different tissues?
- How does the protective action of anti-histone antibodies in animal models of AOI compare to their action in human patients with AOI? Can we potentiate the protective effects of endogenous proteins such as CRP and aPC?

**Figure 1:**
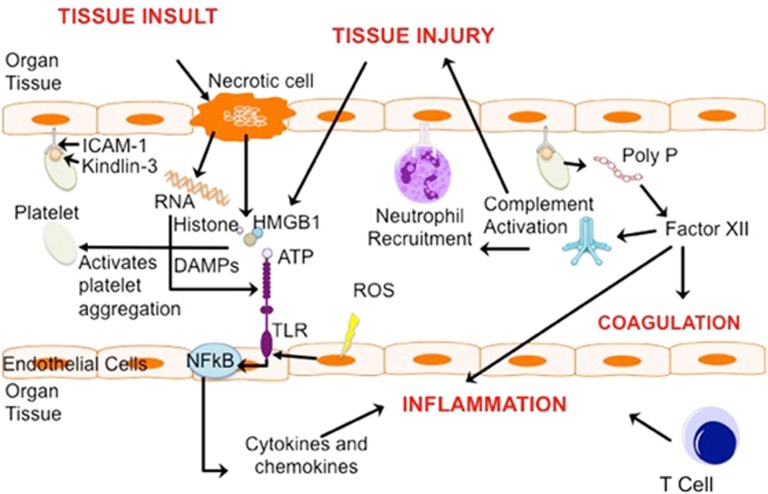
Mechanisms of Sterile Organ Injury. Toxic insults, such as ischaemia or trauma, initiate both controlled and uncontrolled cell death in endothelial cells leading to apoptotic/necrotic tissue and release of intracellular cell components into the extracellular space. These include immunogenic compounds such as RNA and DAMPs (HMGB1, ATP and Histone) which bind to and activate specific TLRs, driving the NFkB-mediated transcription of pro-inflammatory cytokines. TLRs are upregulated by ROS as a result of hypoxic mitochondrial dysfunction. Reperfusion of the tissue and chemokine action results in leukocyte and platelet migration/extravasation. Platelets adhere to the endothelium via ICAM-1 and Kindlin-3. Activated platelets release Poly P, which activates Factor XII, and subsequently, complement. This results in activation of the coagulation pathways and further tissue injury, oedema and inflammation. Activated T cells release pro-inflammatory mediators and can cause direct cytotoxicity

Acute organ injury (AOI) occurs after a toxic insult such as sepsis, trauma, ischaemia/reperfusion (I/R) injury and autoimmune disease.^1, 2, 3^ AOI is characterised by a highly pro-inflammatory environment potentiated by cytokine release, leukocyte migration, microvascular thromboses and cellular death.^2, 4^ ‘Traumatic injury’ activates systemic immune responses, characterised by a pro-inflammatory phase where cytokines, chemokines and damage-associated molecular pattern (DAMP) proteins predominate and an anti-inflammatory immunosuppressive phase.^5^ DAMP proteins, including high-mobility group box 1 (HMGB-1), purines such as adenosine triphosphate (ATP), DNA/RNA and, more recently, histones, have been found to act as immune activating ‘endogenous danger signals’ in these disease states^1^ (Figure 1). I/R develops after a period of interrupted blood flow and lack of tissue perfusion.^1^ A reduction in oxygen delivery and metabolic substrate clearance creates a hypoxic and inflammatory environment, potentiating local necrosis. Current treatments of AOI are mainly supportive. To improve patient outcomes, it is necessary to expand our understanding of the molecular mechanisms of AOI to further aid the development of novel highly targeted and efficacious drugs. This review will describe histone release, their extracellular pro-inflammatory interactions in organ injury and novel approaches for developing histone-targeting drugs.

## Histone biology and function

**Figure 2:**
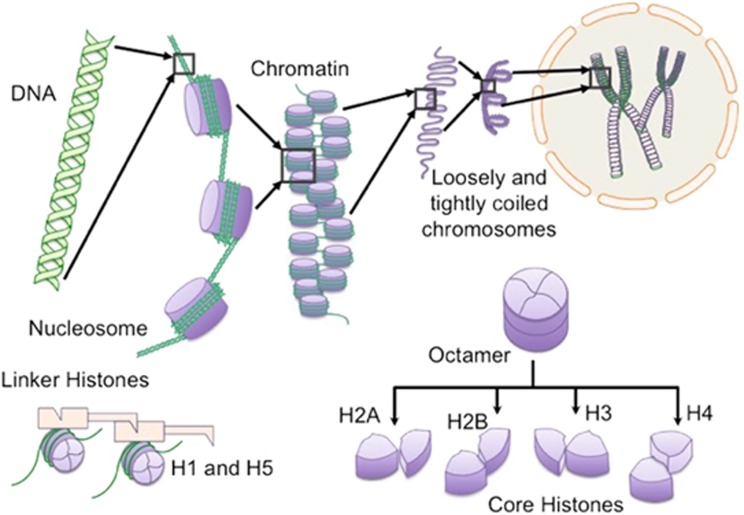
Intracellular structure and function of histone. Histones are intra-nuclear alkaline proteins that contribute to the structural organisation and stability of chromatin. Individual core histone monomers (H2A, H2B, H3 and H4) combine to form octameric structures. Each octamer is made up of two H3-H4 and two H2A-H2B dimers. DNA strands (146 base pairs) wind around the octamers to form nucleosomes and are held together with linker histones (H1 and H5), forming chromatin. Chromatin coils and condenses to form chromosomes. This enables vast amounts of DNA to be compacted tightly within the nucleus of the cell

Discovered in 1884 by Albrecht Kossel,^6^ histones are highly conserved, intra-nuclear, cationic proteins found to have a range of extensively characterised intracellular functions, such as enhancing the structure and stability of chromatin and the epigenetic regulation of DNA.^7, 8, 9, 10, 11, 12^ There are two functional subgroups of histone, ‘core’ (H2A, H2B, H3 and H4) and ‘linker’ (H1 and H5) proteins^13^ (Figure 2). A ‘nucleosome’ is formed of 147 base pairs of DNA wound around an octameric core histone complex. Linker histones join together adjacent nucleosomes and regulate DNA exposure to other intra-nuclear proteins, essential for transcription, replication and repair^7, 8, 13, 14, 15^ (Table 1).

An extensive body of research now demonstrates that extracellular histone is pro-inflammatory.^18^ However, the complexity of this subject is amplified by the existence of two distinct extracellular forms of histone: ‘free histone’ and ‘DNA-bound histone (nucleosomes)’, both differing significantly in their mechanism of cellular release and extracellular interactions.^20^ Crudely illustrated, when intravenously administered to mice, free histone is lethal within minutes,^21^ whereas nucleosome infusions surprisingly produce no immediate cytotoxic effects.^22^ The discriminative discussion within the literature is invariably poor; with many authors failing to clearly differentiate between the two.^20^ It is therefore challenging to accurately interpret the evidence and calls into question the validity of such evidence. This is in part due to the lack of specific anti-histone mAbs that precisely distinguish between free histone and nucleosomic material in serum. To date, the most widely used antibodies, created by Xu *et al.*,^21^ detect H2A-H2B-DNA complexes and antibodies used to identify free histone also interact with histone in nucleosomes. Appraisal of the studies presented within this review is limited by these incongruences. With this in mind, it is vital for future research in this field to focus on accurately delineating the attributes of each distinct form of histone. We have taken care to refer to each form as ‘free histone’, ‘nucleosome’ or ‘NET’ as appropriate within this review.

Free histone and nucleosomes are released from dying cells, particularly during necrosis, which occurs extensively in pathologies precipitating acute organ injury.^23^ ‘Necrosis’ results in an uncontrolled rupture of the plasma membrane, releasing the intracellular contents of a cell including intra-nuclear proteins^4, 24^ Histones are present in the surrounding extracellular milieu after other forms of regulated necrosis including necroptosis, pyroptosis and ferroptosis^10^ and are also expressed at the surface of apoptotic cells.^25, 26, 27^

Neutrophil extracellular traps (NETs) containing histone are released by innate immune cells during a process called NETosis.^28^ This unique form of immune cell death is mediated by protein arginine deaminase 4 (PAD4), an enzyme that facilitates citrullination of H3 and subsequent chromatin decondensation.^29^ Composed of a complex arrangement of antimicrobials, myeloperoxidase (MPO)-bound DNA and histones, NETs have protective and pathogenic functions.^30, 31^ When released from neutrophils during infection, the granule proteins and chromatin form a protective mesh that filters and destroys pathogenic organisms.^30, 31, 32^ However, NETosis also occurs inappropriately during sterile inflammation resulting in thrombosis,^33^ autoimmunity^34^ and NET-mediated cytotoxicity.^35^ It is still unclear what initiates pathological NETosis; however, there is growing evidence that TLR2, TLR4 and complement may have a significant role in initiating this unique process of immune cell death.^36, 37, 38^

**Figure 3:**
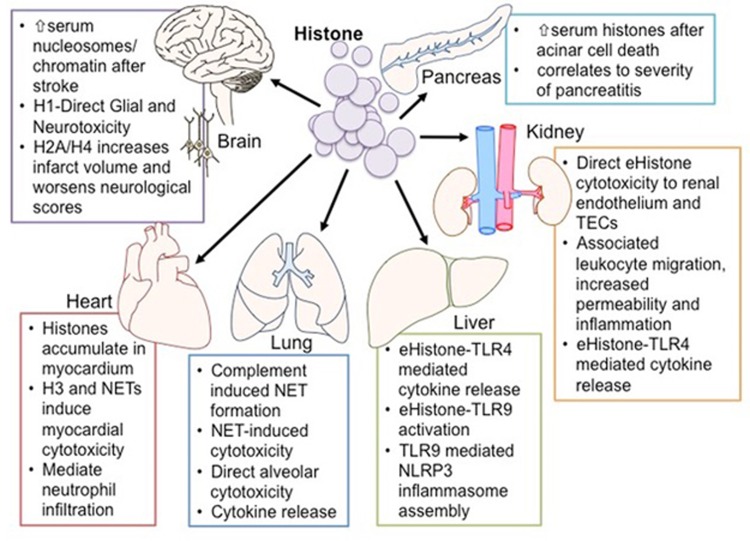
Organ-specific extracellular histone effects. Histone (H) action is diverse, affecting a wide range of cells and tissue types. Common mechanisms include direct cytotoxicity, immune cell TLR stimulation and further immune activation (NLRP3 inflammasome and complement). Intravenous (IV) infusion of histone primarily causes death via alveolar cytotoxicity, before affecting other distal organs. Histone release is correlated with severity of disease and tissue damage

In the extracellular space, histone acts as a DAMP protein (an endogenous danger signal) alerting the body to cellular death, by activating the immune system and repair processes.^39^ In human observational studies, the normal serum value of histone is reported to be ~0.06 ng/ml. However, serum levels as high as 3 ng/ml have been reported in multiple trauma patients^40^ and correlate with coagulopathy, endothelial damage and inflammation, all of which are hallmark features of AOI.^41, 42^ Dynamic serum changes after inflammatory insults could indicate the use of serum histone, nucleosome or NETs a novel biomarker of cell death/NETosis and, hence, disease severity, partly due to the brevity of inflammatory diseases that demonstrate DAMP involvement. DAMPs have been implicated in cancer,^43, 44^ autoimmune disease,^45, 46, 47, 48, 49, 50^ neurodegenerative disease,^51^ sterile inflammation and sepsis.^21, 52^ In 2009, Xu *et al.*^21^ published data demonstrating a significant role for eHistone in driving endothelial dysfunction and organ failure in sepsis. There now exists a vast body of literature that demonstrates histone-mediated pathogenesis in AOI, through activation of TLRs, immunomodulatory effects and the cytotoxic disruption of plasma membrane function (Table 2 and Figure 3).

## Mechanisms of histone-mediated inflammation

**Figure 4:**
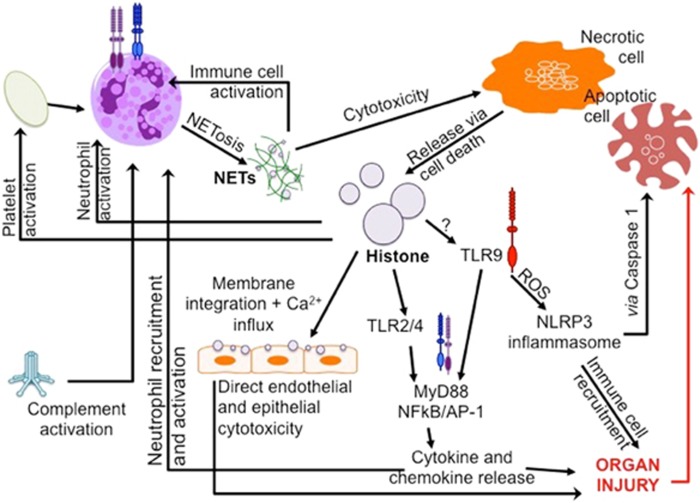
Mechanisms of extracellular histone-induced organ injury. Histone is released from tissue cells through necrotic and apoptotic cell death caused by toxic stimuli, such as ischaemia. Cell membranes degrade, allowing intra-nuclear material and other DAMPs to be released into the extracellular space. Immune cell death (termed NETosis) may also release significant amounts of histone in the form of NETs. Thought to be an antimicrobial component of the innate immune system, NETs also exhibit significant cytotoxicity to tissues and further stimulate immune cell activation. Histone action is independent of its origin, causing damage in distal tissues and organs. Histones cause organ injury via direct endothelial/epithelial cytotoxicity, TLR and complement activation. Histone integrates into the phospholipid bilayer of cell membranes, altering their permeability, resulting in an influx of calcium ions and cell death. Histone-mediated complement activation recruits immune cells and results in further histone release. Histone-activation of TLRs stimulates MyD88-dependent signalling pathways and pro-inflammatory cytokine/chemokine release. Activation of TLR9 causes the release of ROS, activating the NLRP3 inflammasome, Caspase 1 and further inflammatory cell recruitment. These processes converge to cause significant organ injury

*TLRs* facilitate the recognition of invading pathogens by responding to pathogen-associated molecular pattern (PAMP) proteins such as bacterial CpG DNA and lipopolysaccharide.^38, 62, 63, 64^ It is now widely accepted that TLRs also mediate DAMP signalling.^40, 65^ Activation of TLR2 and TLR4 in particular, is likely to be responsible for the release of pro-inflammatory cytokines (IL-6, TNF-α) via MyD88-dependent pathways and the activation of platelets, which drive the augmented immune response in sterile AOI^2, 65, 66, 67, 68^ (Figure 4). Extensive evidence for TLR2 and TLR4 signalling, but not TLR3/5/7/8/9, has been reported. TLR2 and TLR4 KO mouse models are protected from lethal doses of histones;^2, 58^ and TLR2/4 blocking mAbs significantly protect wild-type animals.

However, an additional role has been proposed for the endosomal TLR9 in animal models of hepatic IR injury.^69^ Quantitatively, eHistone-mediated TLR9 activation induces significant pro-inflammatory cytokine (TNFα and IL-6) release and increases necrotic tissue size fivefold.^59^ Anti-H3/H4 neutralising and TLR9 blocking mAbs significantly reduce these pathological changes, which are also attenuated in TLR9 and *myd88* KO mice.

TLR9 activation may further contribute to NLRP3 inflammasome assembly in sterile inflammation.^17^ Classically, the NLRP3 inflammasome responds to microbial stimuli by activating the downstream caspase 1 pathway, generating pro-inflammatory cytokine production and leukocyte recruitment.^70, 71^ Histone-TLR9 activation mediates mitochondrial reactive oxygen species (ROS) production and subsequently activates the NLRP3 Inflammasome in non-parenchymal pro-inflammatory kupffer cells (KCs).^17^ This may be partially attenuated by anti-H3/H4 histone antibody and is absent in TLR9 and *nlrp3* KO mice.

This research is surprising as TLR9 is typically activated by bacterial *CpG-DNA.* It is possible that remnant murine DNA bound to histone within the sample is exerting DNA-TLR9-dependent DAMP effects. eHistone may also enhance DNA-TLR9 signalling, as production of IL-6 significantly increases when co-administered.^59^ Further research should be undertaken to further validate the mechanistic relationship between eHistone and TLR9.

Extracellular histone also causes ‘direct cytotoxicity to epithelial and endothelial tissue’. Administration of high-dose exogenous histone ubiquitously results in a significant reduction in cell viability. Pereira *et al.*^72^ first characterised the ionic binding between cationic histone and anionic phospholipids in 1994. Current theories propose that eHistone binds to phospholipid–phosphodiester bonds, similar to their DNA-binding sites, altering membrane permeability,^19, 21^ and initiating calcium ion influx. Intracellular stores are released, disrupting processes necessary for survival. Interestingly, C-reactive protein (CRP), an endogenous acute-phase protein released during inflammatory diseases and typically used as a biomarker for disease regression, was found to protect against histone-mediated toxicity by binding to phospholipids, blocking histone integration into cell membranes and preventing calcium influx.^73^

## Extracellular histone in acute organ injury

### Brain

Cerebral vascular events are the most common cause of mortality and long-term morbidity across the world. Thrombo-embolism of the cerebral arteries results in acutely reduced cerebral perfusion, and if prolonged, irreversible inflammatory neuronal injury.^74^

*In vivo* animal studies conducted to characterise the release and functional role of extracellular nucleosomes^53^ show that animals exposed to moderate hypoxia (6% over 24 h), demonstrate a threefold rise in circulating levels of nucleosomes compared with control animals. Furthermore, models of ischaemic stroke show a sevenfold serum nucleosome increase. Human observational trials^75, 76^ measuring serum nucleosome after stroke corroborate this evidence, suggesting that poor oxygen perfusion stimulates the release of nucleosomes from cells into the systemic circulation.

Administering exogenous histone or anti-H2A/H4 antibody respectively increases or decreases the infarct size by ~30% when compared with control animals.^53^ The exacerbating effect of histone in these models of IR injury may be due to a combination of factors, including direct endothelial and blood–brain-barrier toxicity,^21, 35, 77^ which increases permeability, leukocyte migration and immune stimulation. In addition, histones activate platelets,^33, 66^ increasing the risk of further ischaemia during in the reperfusion phase.

Patients with neuro-inflammatory conditions, including Alzheimer’s and Parkinson’s, have been reported to have high serum concentrations of histones.^78^ Histone that has leaked into the extracellular space is able to damage glial cells, which support normal neuronal function. It is likely that the immune-derived microglial cells have a key role in the pathogenesis of these pathologies. H1 histone, but, interestingly, not core histones, show dose-dependent neurotoxicity,^54^ by promoting microglial survival. Low doses upregulate major histocompatibility complex class II receptor expression in microglia and demonstrate potent dose-dependent chemoattractant properties. In addition, H1 histone is neuro-immunomodulatory inducing astrocytes to take an activated stellate morphology, increasing their reactivity. These novel findings highlight the mechanistic specificity of histones, whereby the activity status of different cell types is directly affected by their interaction with histone molecules.

### Heart

Cardiac injury is typically caused by I/R disease where a lack of oxygen delivery to cardiomyocytes results in necrosis and release of immunogenic intracellular components.^79^ Repeated ischaemic insults result in irreversible damage and cardiac failure. The prognosis for these patients remains poor despite the recent introduction of disease modifying therapies.

Histones released from necrotic cells accumulate within the myocardium early after myocardial infarction (MI),^55^ inducing further dose-dependent myocardiocyte toxicity. NET-derived nucleosomes are also implicated in driving inflammatory signalling after cardiac ischaemia and are released via NETosis.^56^ Mice lacking a functional PAD4 enzyme produce lower circulating concentrations of nucleosomes post MI. Furthermore, acute treatment with DNase1, an endogenous endonuclease enzyme that disrupts chromatin stability by degrading linker DNA, significantly improves ventricular remodelling, local cardiomyocyte survival, cardiac function and reduces neutrophil infiltration. The observed protective effect of DNase1 treatment is attributed to dispersal of toxic histone molecules, preventing further direct cardiac toxicity. Despite this, no improvements in mortality, infarct size or inflammatory parameters have been observed, and although local cytotoxicity is reduced, immunostimulatory histone is free to disseminate hematogenously, with the potential to damage distal organs.^35^ Despite a relatively small benefit, DNase1 may provide a useful approach to the acute treatment of MI, whereby small amounts of the myocardium may be preserved and prolong cardiac function for high-risk patients.

### Lung

The mortality rate associated with acute lung injury (ALI) is ~40%.^80^ ALI may arise secondary to transfusion, trauma or ischaemia, and affects normal alveolar and pulmonary endothelial cell functioning, resulting in inefficient gas exchange, increased protein permeability, cell death, inflammation and eventually, permanent lung dysfunction.^81, 82^

Animal models of transfusion-related lung injury (TRALI) demonstrate significant NET-mediated inflammation.^57^ Activated platelets induce NET formation from neutrophils and, once in the extracellular space, NETs increase pulmonary oedema and vascular endothelial permeability.^35^ It is unclear whether the histone, DNA or granular proteins within NETs, or indeed a combination of components, are responsible for mediating pulmonary cellular death and inflammation. It is likely that all three components have a part to play; however, Caudrillier *et al.*^57^ noted a significant reduction in alveolar cell viability upon administration of pure histones *in vitro.* Furthermore, pre-incubation with anti-histone (H1-DNA, H2A, H2B and H4) antibody significantly decreases NET-mediated cytotoxicity in TRALI-mice. The endogenous anionic glycan, polysialic acid (PSA) is also protective in this model and works by binding cationic histone directly, an interaction, which was previously linked to neural regeneration in cerebral tissue.^35, 83^ Interestingly, the seemingly inert properties of free H3 in alveolar tissue is also described by Saffarzedeh *et al.*^35^ While infusion of H1 and H4 induce alveolar cell death, H3 does not.^36^ In addition, the use of anti-H3/citH3 mAb confers no further protection in models of ALI,^35^ whereas anti-H4 IgG antibody significantly attenuates pro-inflammatory signalling, reducing ALI severity by 50%.^36^ These differences highlight the importance of conducting similar experiments in other tissues in order to elucidate the specific cytotoxic activities of H1-5.

Acute lung pathology may also frequently occur secondary to major traumatic events.^84^ Pathologically, major trauma results in pulmonary leukocyte infiltrates, tissue oedema, microvascular haemorrhage and thrombosis.^85^ In patients with an acute history of severe blunt trauma, circulating histones increase to toxic levels within 4 h and correlate with the severity of lung injury, endothelial damage and coagulation. Pathological changes observed in mouse models of trauma-associated lung injury include neutrophil-obstructed alveolar capillaries, pulmonary haemorrhage and tissue oedema.^19^ Immunohistochemical staining for histone *in vitro* and *in vivo* shows accumulation around endothelial cell membranes where they interact with phosphodiester bonds on phospholipids, which are similar to DNA-histone-binding sites.^72, 86^ The histone–phospholipid interaction alters membrane permeability and results in calcium influx-dependent cell death.^19^ Similar findings are also reported in other tissue types, including platelets, breast cancer cells, urinary bladder epithelium and thymocytes.^65, 66, 87, 88, 89, 90^ This discovery is fundamental to the understanding of the nonspecific, TLR independent, cytotoxic effects of free histone.

### Liver

Hepatic ischaemia and drug toxicity are forms of sterile hepatitis that can result in liver enzyme derangement and synthetic function disturbance presenting as encephalopathy, coagulopathy (reduced clotting factors) and oedema (low albumin),^91^ all of which are hallmark attributes of acute liver failure (ALF).

Histone is released after hepatic sterile inflammation and activates TLRs on KCs, initiating a cytokine storm.^2, 58^ However, in contrast to the aforementioned models of ALI, the release of *H3* into the ‘hepatic circulation’ is associated with significant hepatic damage that is attenuated in the presence of anti-H3 antibody, which reduces the risk of mortality and serum measurements of TNF-α and IL-6 in animal models of ALF. In addition there is controversy surrounding the role of TLR2, TLR4 and TLR9 in mediating inflammation in I/R animal models. This is discussed in detail in the previous section entitled ‘Mechanisms of histone-mediated inflammation’.

NETs also mediate hepatocytotoxicity.^60^ Initially, NETosis is stimulated by histone activation of TLR4 and TLR9 expressed on neutrophils (Figure 4). NETs stimulate immune-derived KCs to release pro-inflammatory mediators, which is abolished upon co-administration of NETosis inhibitors, PAD4i or DNase1. A reduction in both the histone-mediated release of NETs and the markers of hepatic injury is observed.

### Kidney

Acute ischaemic kidney injury is a common clinical complication and carries a high rate of long-term morbidity and mortality.^92^ Ischaemic renal damage is characterised by apoptotic and necrotic cell death, and perpetuated by a rise in local pro-inflammatory cytokines and invading neutrophils.^93^ Repeated insults to the renal parenchyma can result in permanent fibrosis and renal dysfunction, which, if left untreated, may progress to chronic kidney disease.^94^

**Figure 5:**
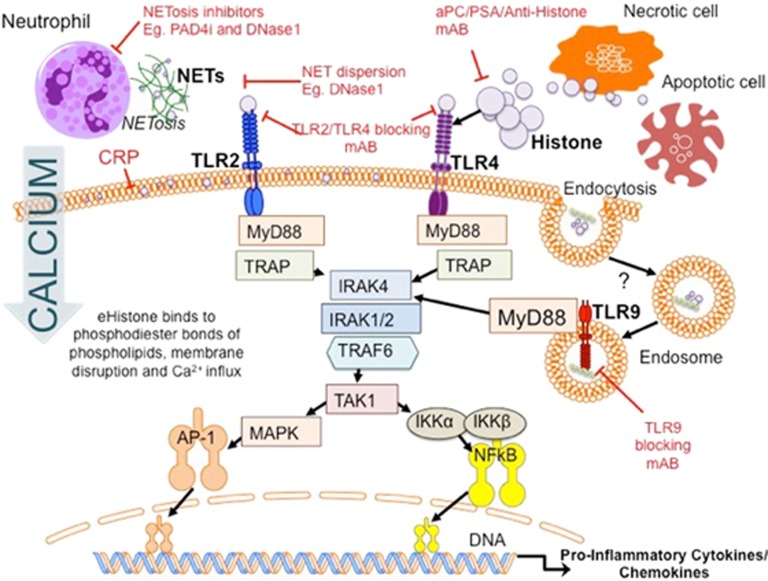
eHistone signalling via TLRs and potential therapeutic approaches. Histones are released via NETosis and cellular death mechanisms during organ injury and bind with TLRs. This activates the MyD88-dependent signalling pathways and results in the transcription and translation of pro-inflammatory cytokines and chemokines, including TNFα, IL-6. Several therapeutic approaches are proposed, including those that target eHistone directly such as activated protein C, polysialic acid and anti-histone antibody; those that block TLR receptor signal transduction and those that prevent eHistone release by reducing apoptotic and necrotic cell death

During acute renal ischaemia, histone is released into the extracellular space by necrotic tubular epithelial cells.^16^ eHistone induces dose-dependent toxicity to renal endothelium and tubular epithelium, leukocyte adhesion, increased vascular permeability and transendothelial migration within the renal arteries. Neutralisation of histone activity using anti-H4 mAb reduces cytokine and chemokine expression.^95^ It is highly likely that TLR2 and TLR4 mediate and activate pro-inflammatory MyD88-NFkB and MAPK pathways (Figure 5) and corroborating studies have shown that these pro-stimulatory effects are attenuated in MyD88 and TLR double-knockout animals.^68, 96^

### Pancreas

Ischaemic damage to the pancreas can occur after very brief episodes of reduced pancreatic perfusion and is characterised by rapid release of pancreatic enzymes and severe tissue inflammation.^97^ Commonly occurring after cardiac surgery, the pathology can progress to acute pancreatitis when acinar cell death occurs.^98^

During gallstone and cholecystokinin-induced necrotising pancreatitis,^61^ necrotic acinar cell death predominates with large volumes of histone released into the extracellular space. Oedema and inflammation alone does not result in a serum histone rise and it is likely that release of histone from acinar cells in pancreatic injury is a necrosis-dependent mechanism. However, histone may also be released during pancreatitis due to a lack of HMGB-1, which is protective against oxidative stress and DNA disruption.^99^ Once in the extracellular space, histone promotes HMGB-1 secretion from innate immune cells, further accelerating DNA damage in these cells by reducing the intra-nuclear concentration.^100^ Research showing the benefit of anti-H3 mAbs is promising in these models;^99^ however, the discussion of histone action in pancreatic injury is limited within the literature and further research is required to fully confirm the suspected involvement of TLR pathways involved in sterile pancreatic injury.

## Novel approaches for targeting histone

Extracellular histone and nucleosome levels are correlated with poorer outcomes in human observational studies. There exists a theoretical potential to reduce the burden of AOI by blocking their extracellular actions. We propose three therapeutic strategies for attenuating the deleterious effects of histone (Table 3). They include blocking the release of histone, neutralising circulating histone and blocking eHistone signal transduction.

Targeting the release of histone complexes and, in particular, inhibiting NETosis has demonstrated considerable specificity and efficacy when used in animal models of AOI^55, 56, 60^ and sepsis.^101^ Specific PAD4 inhibitors prevent the citrullination of H3, a key step in releasing nucleosomic material for NET formation,^102^ and are more effective than DNase1 in preventing tissue damage.^103^ DNase1 theoretically disperses DNA-bound-histone, and hence, reduced NET-mediated cytotoxicity.^55^ but are unlikely to be able to effectively access DNase1 binding sites within multifarious NET complexes.

Drugs targeting histone directly must only act in the extracellular space. Histone neutralising drugs that obtain intracellular access could potentially disrupt DNA structure or function, and result in catastrophic side effects. Interestingly, plasma concentrations of endogenous proteins such as activated protein C (aPC) and CRP inversely correlate with serum histone^40^ suggesting that histone action may be partially attenuated by the action of these molecules.^104^ APC is a serum protease, which degrades histone in the circulation and although a systematic review of five randomised control trials (RCTs; *n*=5101) demonstrated no benefit of using aPC in patients with severe sepsis,^105^ studies using recombinant aPC have shown significant protection in animal models of sterile inflammation.^106^ Similarly, the endogenous, acute-phase protein CRP protects against histone toxicity by preventing coagulation activation, inhibiting endothelial damage and reducing vascular permeability in murine models infused with histone.^73^ In addition, histone-CRP complexes are observed in the serum of patients and CRP is able to compete for phospholipid-binding sites, preventing histone integration into plasma membranes, calcium influx and cell lysis. Enhancing the activity of these proteins may provide a promising avenue for the future treatment of inflammatory diseases.

Despite their use in laboratory experiments, TLR blocking mAbs are unlikely to provide a promising direction for anti-histone drugs. Their intrinsic action of eliciting innate antigen-specific acquired immunity after host infection^64^ would mean that blocking their activity might cause substantial immunodeficiency. However, there is considerable evidence for the protective effects of histone neutralising mAbs in animal models of AOI.^2, 16, 17, 19, 21, 35, 36, 53, 57, 58, 59^ The data show an improvement in inflammation, functional scores and overall survival. This is encouraging and paves the way for future development of drugs with similar pharmacodynamic properties for human use in inflammatory pathology.

## Future perspective

Future research in this field should focus on developing an accurate means of detecting free histone. This would significantly improve the validity of similar work and further elucidate the specific molecular interactions of histone *in vivo* compared to those currently attributed to NETs and nucleosomes. In addition to this misnomer, there is limited discussion within the literature describing the differences in cytotoxic activity of H1-5. Although Saffarzedeh *et al.* attempts this by presenting H1-H5 effects individually,^36^ these experiments only represent a single cell type and are not representative of their action in other models of AOI.

## Conclusion

It is clear that both free and DNA-complexed histone have important roles in mediating pro-inflammatory signalling in sterile AOI. Released during periods of cell death and immune activation, histone, nucleosomes and NETs induce cytotoxicity by altering cell membrane permeability to calcium ions, activating TLRs on innate immune cells, stimulating NLRP3 inflammasome and complement systems, resulting in a sterile pro-inflammatory environment.

There are three distinct pharmacodynamic approaches to target histone-mediated inflammation, by reducing the release, neutralising or blocking histone signal transduction. Although these approaches have proven to provide significant protection from mortality in animal models of acute organ injury, further research is necessary to warrant their safe application in a clinical setting.

## Ethical considerations

The authors declare that no ethical considerations were highlighted during the writing of this report.

## Figures and Tables

**Table 1:** Functions of histone

**Intracellular functions**	**Extracellular functions**
Nucleosome construction^7^	DAMP signalling via TLR2 and TLR4 receptors^16^
Chromatin stability^7^	NLRP3 inflammasome activation^17^
Epigenetic modifications of transcription, replication and repair of DNA^15^	Cell mediated apoptosis^18^
	Neurogenesis, migration and endocytosis^18^
	Direct cellular toxicity^19^
	Regulation of inflammation, immunity, death, coagulation and thrombosis^18^

**Table 2:** Organ-specific effects of extracellular histone in various models of sterile organ injury

**Organ**	**Animal model of AOI**	**Effects of histone/anti-histone therapy**
Brain	I/R^53^	Chromatin released post I/R injury
		Exogenous histone infusion
		Increases infarct volume
		Worsens neurological scores.
		Improve neurological scores
		Anti-H2A/H4 antibodies
		Reduce infarct volume
	Histone-induced toxicity^54^	Dose-dependent, H1 neurotoxicity
		H1-mediated microglial
		Survival
		Increased reactivity
		MHC class II receptor expression
		Chemoattractant activity
Heart	I/R^55, 56^	Accumulation of eHistones within myocardium
		eHistone-mediated myocytoxicity
		PAD4 KO mice are protected from MI injury
		DNase1 treatment
		Improves ventricular remodelling
		Prolongs local cardiomyocyte survival
		Reduces MI volume
		Improves cardiac function
		Reduces nucleosome release and neutrophil infiltration
		No effect on mortality, infarct size or inflammation
Lungs	TRALI^57^	Activated platelets promote NET formation in TRALI
		NETs increase permeability of LPS primed endothelial cells
		Anti-H2A/H4 antibodies attenuate
		Histone-mediated lung oedema
		Vascular permeability
		Mortality
		Prevent further NET formation
	NET induced^35^	NETs and eHistones induce cell death
		Epithelial
		HUVECs
		DNase
		Does not decrease NET-mediated cytotoxicity
		Anti-H1/2A/2B/4 antibodies, PSA and APC are protective
	IgG and Complement induced^36^	eHistone
		Released into BALF of ALI patients
		Dependent on complement (C5aR and C5L2) activation
		Exhibits alveolar epithelial cytotoxicity
		C5aR and C5L2 activation induces neutrophil-dependent ALI (?NETs)
		Anti-H4 IgG antibody attenuates ALI severity
	Trauma^19^	Serum histone reaches toxic levels post-trauma
		Release correlates with
		Lung injury severity
		Endothelial damage
		Coagulation
		eHistone actions
		Direct toxicity to endothelial cells
		Stimulate cytokine and NET release
		Phospholipid-histone complexes result in direct cellular toxicity through membrane disruption and calcium influx
Liver	ConA and APAP induced^2^	eHistones stimulate a “cytokine storm”
		Potentiate TLR2 and TLR4 signal transduction
		No activity at TLR3/5/7/8/9
		Cytokine release is abolished in TLR4 KO mice
		H3 histone is released in ConA and APAP induced liver injury
		Anti-H3 antibody
		Reduces mortality and cytokine release
		Does not prevent histone release or improve liver injury markers
	I/R^17, 58, 59, 60^	Histones released from hepatocytes post-I/R injury
		Histone infusion
		Worsens markers of acute liver injury
		Activates non-parenchymal KC TLR9-MyD88 pathways
		Enhances DNA-TLR9 signalling
		TLR9-mediates mitochondrial ROS production
		ROS activates NLRP3 Inflammasome
		Effects attenuated in TLR9 and NLRP3 KO mice
		DAMPs (eHistone and HMGB1) stimulate NET formation post I/R injury by activating TLR4 and TLR9
		NETs
		Hepatocytotoxic
		Stimulate KC-cytokine release
		Formation is inhibited by PAD4i and DNase1
		Anti-H3/H4 histone antibody
		Attenuates TLR9 signalling
		Improves markers of acute liver injury
Kidney	I/R^16^	Necrotic TECs release histone
		eHistone actions
		Direct toxicity to renal endothelial and TECs
		Leukocyte recruitment
		Microvascular vascular leakage
		Renal inflammation
		Activates TLR2/TLR4 and potentiates NFkB, MyD88, MAPK signalling
		Anti-histone IgG is protective
Pancreas	Gallstone and CCK^61^	Histone released from necrotic acinar cells.
		eHistone concentration correlates well with severity of tissue injury

Abbreviations: APC, activated Protein C; ALF, acute liver failure; ALI, acute lung injury; APAP, Paracetemol/Acetominophen; BALF, bronchoalveolar lavage fluid; C5aR, component 5a receptor; C5L2, anaphylatoxin chemotactic receptor; citH3, citrullinated H3; ConA, Concanavalin A; DNA, deoxyribonucleic acid; DNase, deoxyribonuclease; EC, endothelial cells; eHistones, extracellular histones; ELISA, enzyme linked immunosorbent assay; H1/H2A/H2AX/H2B/H3/H4/H5, histone subtypes; HMGB1, high-mobility group box 1; HUVEC, human vascular endothelial cells; IgG, immunogloblin G; KC, kupffer cells; KO, knockout; I/R, ischaemia reperfusion; LPS, lipopolysaccharide; mAb, monoclonal antibody; MAPK, mitogen-activated protein kinases; MCA, middle cerebral artery; MHC, major histocompatibility complex; MI, myocardial Infarction; MPO, myeloperoxidase; MyD88, myeloid differentiation primary response gene 88; NET, neutrophil extracellular traps; NFkB, nuclear factor kappa B; NLRP3, nucleotide-binding domain leucine-rich repeat containing protein 3; NS, non-significant result; PAD4i, peptidyl-arginine-deiminase-4; PSA, polysialic acid; ROS, reactive oxygen species; S, significant result; TECs, tubular epithelial cells; TLR, Toll-like receptor; TRALI, transfusion-associated lung injury

**Table 3:** Therapeutic approaches and potential anti-histone therapies

**Blocking release**	**Neutralisation**	**Blocking signalling**
PAD4i^60^	Anti-H mAb^2, 16, 19, 35, 36, 53, 57, 58, 59^	TLR blocking mAb
Blocks NETosis	Neutralises histone in circulation	PreventsTLR2/4/9 signal transduction
Inhibits citrullination of H3	DNase1^55^	Reduces cytokine release
DNase1^57^	Degrades NET linker-DNA	Significant immunosuppressive side effects likely
Disperses NET-derived histone within circulation	Disperses histone	CRP^73^
Prevents NET-mediated NETosis	aPC^35^	Endogenous anionic acute phase protein
	Serum protease	Prevents cationic histone from binding to phosphodiester bonds on phospholipids
	Degrades eHistone	
	PSA^35^	
	Endogenous Anionic protein	
	Ionic interaction with H	

Abbreviations: aPC, activated Protein C; BWA3, H4 histone neutralising antibody; CRP, C-reactive protein; H, histone; NET, neutrophil extracellular trap; NETosis, NET release; PAD4i, protein arginine deiminase 4; PSA, polysialic acid; TLR, Toll-like receptor
